# Characterization of *Hoplolaimus seinhorsti* and *Hoplolaimus pararobustus* (Tylenchina: Hoplolaimidae) from banana, with phylogeny and species delineation in the genus *Hoplolaimus*

**DOI:** 10.2478/jofnem-2023-0019

**Published:** 2023-05-23

**Authors:** Emmanuel Olajide, Phougeishangbam Rolish Singh, Yao A. Kolombia, Merlin Kornelia Rumbarar, Marjolein Couvreur, Wim Bert

**Affiliations:** 1 Nematology Research Unit, Department of Biology, Ghent University, K.L. Ledeganckstraat 35, 9000 Ghent, Belgium; 2 International Institute of Tropical Agriculture (IITA), Head Quarters and West Africa Hub, P.M.B. 5320, Oyo Road, Ibadan 200001, Oyo State, Nigeria; 3 Papua Assessment Institute for Agricultural Technology, Jl. Yahim Sentani, PO box 256, Jayapura 99352, Papua, Indonesia.

**Keywords:** 28S rDNA, ITS rDNA, *COI*, Banana, *Hoplolaimus*, Lance nematode, Morphology, Phylogeny, Plant-parasitic nematode, Scanning electron microscopy, Systematic, Taxonomy

## Abstract

The morphological and molecular characterisations of two lance nematode species isolated from the rhizosphere of banana, *Hoplolaimus seinhorsti* and *H. pararobustus*, are provided based on an integrative study that includes light and scanning electron microscopy, phylogenetic analysis and two tree-based molecular species delimitation methods (GMYC and bPTP). Nineteen new sequences were obtained, including 5 partial 18S rRNA, 6 D2-D3 of 28S rRNA, 1 ITS rRNA and 7 *COI* mtDNA (the first *COI* sequences of *H. seinhorsti* and *H. pararobustus*), and an updated morphological character comparison of 37 *Hoplolaimus* species is presented. The tree-based molecular species-delimitation approaches employed gave markedly differing results, and also showed remarkable discrepancies among the investigated genes, although the bPTP output was found to agree well with established morphological species delimitations. Both species-delimitation approaches did, however, provide the same output for the *COI* mtDNA sequences, and the *COI* mtDNA gene sequence was also found to correspond better to established morphological species. It is therefore recommended by this paper as representing the most suitable barcode marker for *Hoplolaimus* species identification. This integrative study also resulted in the corrective reassignment of 17 gene sequences that were previously unidentified or incorrectly classified, as well as concluding that *H. pararobustus* consists of two cryptic species.

The genus *Hoplolaimus* Daday, 1905, subfamily Hoplolaiminae Filipjev, 1934 and family Hoplolaimidae Filipjev, 1934 was established based on a single female of *Hoplolaimus tylenchiformis* Daday, 1905 from Paraguay (Sher, 1963). To date, 37 valid species within this genus have been described (Handoo and Golden, 1992; Tiwari et al., 2001; Ali et al., 2009; Nguyen et al., 2015; Ma et al., 2019; Ghaderi et al., 2020). The descriptions of these species are primarily based on morphology and morphometrics alone, as molecular data are unavailable for all but for ten of these *Hoplolaimus* species. Commonly referred to as lance nematodes, species of the genus *Hoplolaimus* are known for their well-developed and robust stylet with ‘tulip-shaped’ knobs. These plant-parasitic nematodes represent an important group of semi-endo to endo-parasitic nematodes that cause considerable damage to the host plant’s cortex and vascular tissue as well as inducing root necrosis. They are widely-distributed worldwide, and target a broad range of host plants including cotton, citrus, sugarcane, mango, tamarind, cowpea, baobab tree, banana, corn and soybean (Sher, 1963; Bridge, 1973; Robbins et al., 1987, 1989; Henn and Dunn, 1989; Koenning et al., 2004; Ahmadi et al., 2016; Holguin et al., 2015). The infective stages of *Hoplolaimus* species are from J2 to adult (Bridge and Starr, 2007). Some species reproduce sexually (amphimictic), while others reproduce asexually with females producing offspring without fertilization (parthenogenetic) (Bae et al., 2008).

The identification of *Hoplolaimus* species is facilitated by the key proposed by Handoo and Golden (1992), which was developed using information resulting from a comparative study of 29 valid *Hoplolaimus* species. The species-informative morphological characters include, among others, the number and the pattern of lateral field incisures, the number of labial annuli, the position of scutella along the body, the number of esophageal gland nuclei, and the hemizonid position with respect to the secretory-excretory (SE) pore. Ghaderi et al. (2020) recently published a comprehensive book on the systematics of the genus *Hoplolaimus*, in which they presented an updated key for *Hoplolaimus* species identification. This new key incorporates a broader range of informative morphological characteristics compared to the earlier key proposed by Handoo and Golden (1992). However, Bae et al. (2008, 2009) have previously indicated that a relatively large intraspecific variation *vs* minor interspecific variation of the diagnostic characters renders the identification of *Hoplolaimus* species difficult when based solely on morphology and morphometrics, leading them to develop molecular identification tools for some *Hoplolaimus* species from the USA based on duplex and multiplex PCR and PCR-RFLP Prior to Bae’s work on *Hoplolaimus*, several authors had already deposited sequences of genes from this genus in GenBank, including the partial 18S rRNA from *H. galeatus* (AY912054, AY912053) and *H. columbus* (AY912052, AY912051) by Powers, et al. (2008) and *H. galeatus* (AY146452) by Mullin et al. (2005). In addition, Subbotin et al. (2008) sequenced the D2-D3 of 28S rRNA from *H. seinhorsti* (DQ328752) and Chen et al. (2006) deposited partial 18S rRNA gene and complete ITS1-5.8S-ITS2 sequence with partial 28S rRNA from *H. columbus* (DQ309584) in GenBank. Additionally, Powers et al. (1997) reported that some congeneric *Hoplolaimus* species exhibit ITS size length variation and this variation can affect the accuracy of identifying *Hoplolaimus* species based solely on molecular methods that rely on this region.

The aims of this current study were to (1) characterise *Hoplolaimus pararobustus* and *H. seinhorsti* from the rhizosphere of banana *(Musa* spp.) in Nigeria and Indonesia, respectively, based on morphology (light microscopy and scanning electron microscopy) and molecular data (partial 18S rRNA, D2-D3 expansion segment of 28S rRNA, ITS rRNA and *COI* mtDNA sequences); (2) investigate the phylogenetic relationships of the *Hoplolaimus* species in combination with tree-based species-delimitation; and (3) update the existing morphological comparison of the 37 known *Hoplolaimus* species.

## Materials and Methods

*Soil sampling and nematode extraction:* Bulk soil samples were collected using a shovel around the upper 20-30 cm rhizosphere of banana (*Musa* sp.) from Jayapura, Papua (2°40’47.5”S latitude, 140°49’20.9”E longitude), and from the rhizosphere of banana (*Musa* sp.) plant in Onne, Rivers State, Nigeria (4°42’57.7”N latitude, 7°10’34.0”E longitude). The soil samples were stored at 4°C until nematode extraction. Live nematodes (mixed stages) were extracted using the modified Baermann’s method (Whitehead and Hemming, 1965).

*Morphological analysis:* Morphological and morphometric characterization of the two nematode species was conducted based on fresh and fixed specimens. For the preparation of permanent slides, a small suspension of nematodes in an embryo dish were killed and fixed by adding a few drops of Trump’s fixative (2% paraformaldehyde, 2.5% glutaraldehyde in 0.1M Sorenson buffer (Sodium phosphate buffer at pH = 7.5)). Subsequently, the embryo dish was heated in a microwave (700 Watts) for about 5 sec, left to rest for 1 h at room temperature followed by 24 h at 4°C to ensure maximum penetration of the fixative as described in Singh et al. (2018). Afterwards, the nematodes were gradually transferred to anhydrous glycerin for permanent slides following the protocol of Seinhorst (1959) and mounted on glass slides, for further morphological study. Nematodes were examined, photographed, and measured using an Olympus BX51 DIC Microscope (Olympus Optical, Tokyo, Japan) equipped with an Olympus C5060Wz camera. Scanning electron microscopy (SEM) was performed for *H. seinhorsti* specimens (n = 3) fixed in Trump’s fixative, washed in 0.1M phosphate buffer (pH = 7.5), dehydrated in a graded series of ethanol solutions and critical-point-dried with liquid CO_2_. The specimens were mounted on stubs with carbon tabs (double conductive tapes), coated with gold of 25 nm and photographed with a JSM-840 EM (JEOL) at 12 kV (Singh et al., 2018). The *H. pararobustus* population was compared with lectotype and paralectotype material of the Ghent University Museum, Zoology Collections, Belgium (UGMD 100061-63).

*Molecular analysis:* Nematode morphological vouchers were prepared prior to DNA extraction. These vouchers were made of LM pictures of individual nematodes in temporary slides with distilled water. Each nematode was subsequently removed from the temporary mount and cut into pieces in distilled water using a blade and the pieces were transferred to a PCR tube with 20 μl of worm lysis buffer (50 mM KCl, 10 mM Tris at pH = 8.3, 2.5 mM MgCl_2_, 0.45% NP 40 (Tergitol Sigma), 0.45% Tween 20). The PCR tube was then incubated at -20°C (10 min) followed by adding 1μl proteinase K (1.2 mg/ ml), incubation at 65°C (1 h) and 95°C (10 min) and ending by centrifuging the mixture at 14000 rpm for 1 min (Singh et al., 2018). PCR amplification of partial ITS and 18S regions of rDNA was conducted using the primer pairs Vrain2F: 5’-CTT TGT ACA CAC CGC CCG TCG CT-3’ / Vrain2R: 5’-TTT CAC TCG CCG TTA CTA AGG GAA TC-3’ (Vrain et al., 1992) and SSU18A: 5’-AAA GAT TAA GCC ATG CAT G-3’ / SSU26R: 5’-CAT TCT TGG CAA ATG CTT TCG-3’ (Mayer et al., 2007) with thermal profile described in Singh et al. (2018, 2019). For amplification of the D2D3 expansion segment of the 28S rDNA sequence, the primer pair 391: 5’-AGC GGA GGA AAA GAA ACT AA-3’ / 501: 5’-TCG GAA GGA ACC AGC TAC TA-3’ was used as described in Nadler et al. (2006) and for the amplification of the *COI* region of mtDNA, the primer pair JB3: 5’-TTT TTT GGG CAT CCT GAG GTT TAT-3’ / JB4.5: 5’-TAA AGA AAG AAC ATA ATG AAA ATG-3’ was used as described in Derycke et al. (2010). The PCR products were enzymatically cleaned with alkaline phosphatase (1 U/ml) and exonuclease I (20 U/ml) for 15 min at 37°C, followed by 15 min at 8°C and sent for sequencing at Macrogen (https:// dna.macrogen.com).

*Phylogenetic and species delimitation analysis:* The construction of a supermatrix for the phylogenetic analysis of *Hoplolaimus* species was not possible due to the limited availability of relevant sequence data on GenBank. At the time of writing, only a few species were associated with both nuclear and mitochondrial sequences. Therefore, each genetic marker was analysed separately.

The phylogenetic relationship of *H. seinhorsti* with other related species was analyzed based on the D2-D3 of 28S and ITS of rDNA and partial *COI* sequences of mtDNA, while that of *H. pararobustus* was analyzed based on the partial sequences of 28S and 18S of rDNA and partial *COI* sequences of mtDNA.

All sequences were analysed using a suite of programs implemented in Geneious 10.0.9 (https://www.geneious.com). The newly generated sequences were first subjected to a Basic Local Alignment Search Tool (BLAST) search against a closely related set of species on GenBank to identify and collect homologous sequences for multiple sequence alignment and phylogenetic analysis. Multiple sequence alignments were constructed using MUSCLE with default parameters. The poorly aligned regions were manually trimmed to obtain high-quality alignments for subsequent analysis. Bayesian inference was performed using MrBayes 3.2.6, with the general time reversible substitution model and estimation of invariant sites, assuming a gamma distribution with four categories gene (GTR + I + G) model. The analyses were run under 1 × 10^6^ generations with four independent chains to ensure convergence and to obtain the posterior probabilities for the phylogenetic tree. Convergence of the runs was also checked using Tracer v1.7.2 (Rambaut and Drummond, 2010), and the effective sample size (ESS) values were well above 200 (>3000) for each run, indicating that the chains had converged and that the results were reliable. The Markov chains were sampled at every 100 generations, and 20% of the converged runs regarded as burn-in (Huelsenbeck and Ronquist, 2001).

Molecular species-delimitation of *Hoplolaimus* spp. in this study was performed using two tree-based methods, a Bayesian implementation of the Poisson tree processes (bPTP; Zhang et al., 2013) and the generalized mixed-yule coalescent (GMYC; Pons et al., 2006). For the bPTP approach, the phylogenetic trees created by MrBayes were uploaded to the online server of bPTP (http://species.h-its.org/ptp/) excluding outgroups, with default parameters. For the GMYC analysis, ultrametric trees were constructed using BEAST v1.10.4 (Drummond et al., 2012). Strict clock model with a lognormal distribution for the clock rate prior, a Speciation: Yule process for the tree prior, and a Hasegawa-Kishino-Yano (HKY) Substitution Model rate prior were used, and analyses were run for 1 × 10^7^ generations, saving trees every 1 × 10^3^ generations. The final trees were produced after removing 2000 samples (20%) as burn-ins, and the maximum clade credibility tree was calculated using TreeAnnotator 1.10.4. Finally, GMYC species delimitation was performed using a python re-implementation of the single threshold GMYC model in the GitHub repository (https://github.com/iTaxoTools/GMYC-pyqt5), using TreeAnnotator trees as input.

## Results

Handoo and Golden (1992) have previously tabulated a comparison of the various important morphological characters and morphometrics of 29 *Hoplolaimus* species, resulting in an identification key for the species. Entries for eight more *Hoplolaimus* species have been added to this table as a result of this study and Ghaderi et el. (2020) (*H. diadematus*, *H. igualaensis, H. intermedius, H. johani, H. maggentii, H. smokyensis, H. puriensis* and *H. bachlongviensis;* Table 2).

**Table 2.** Comparison of 37 *Hoplolaimus* species after Handoo and Golden (1992) and Ghaderi et al. (2020). The comparison includes eleven morphological and morphometric data and the presence or absence of male. Species included in this study are highlighted in bold and the measurements of body length (mm), stylet length and spicule length are in μm in range.

	Length	Lateral incisures	Gland nuclei	Stylet length	Labial annules	Longitudinal striae on basal lip annules	EP in relation to hemizonid	Intestinal post-rectal sac	Phasmids in relation to vulva	Tail annules	Males	Spicule length
*H. abelmoschi*	1.5-1.8	2	3	42-47	5	25-28	Anterior	Present	Both adjacent, one anterior & one posterior	9-15	Present	44-47
*H. aegypti*	1.3-1.9	1	5-6	45-50	4	13-22	Anterior	Present	One anterior & one posterior	17-27	Present	54-65
*H. aorolaimoides*	0.8-0.9	4	3	31-35	4-5	6-13	Posterior	Present	One anterior & one posterior	6-17	Present	31-37
*H. bachlongviensis*	1.2-1.5	1	6	44-50	4.0	6	Anterior	Absent	One anterior & one posterior	9-13	Unknown	-
*H. californicus*	1.1-1.7	4	3	46-53	6	36	Posterior	Present	Both posterior	14	Present	45-55
*H. capensis*	1.6-2.1	2	3	46-58	5-6	Unknown	Anterior	Present	One anterior & one posterior	9-16	Present	51-70
*H. casparus*	1.2	0	3	39.7	3	Unknown	Anterior	Absent	One anterior & one posterior	12	Present	39-40
*H. caudifurcatus*	1.0-1.1	-	>4	52-55	4-5	Unknown	Anterior	Unknown	Unknown	15-20	Unknown	-
*H. cephalus*	1.2	0	6	34	Smooth	0	Anterior	Absent	One anterior & one posterior	6	Present	33-38
*H. chambus*	1.2-1.6	Breaks	6	41-45	3	6	Anterior	Present	One anterior & one posterior	9-13	Unknown	-
*H. citri*	0.8-1.3	0	6	35-37	4	10-12	Anterior	Absent	One anterior & one posterior	12-15	Present	38-47
*H. clarissimus*	1.4-1.8	4	6	46-53	4	18-31	Posterior	Present	One anterior & one posterior	20-26	Present	55-61
*H. columbus*	1.3-1.8	1	6	40-48	3	10-15	Anterior	Present	One anterior & one posterior	16-22	Rare	37-53
*H. concaudajuvencus*	1.1-2.0	4	3	50-57	5-6	36	Posterior	Absent	One anterior & one posterior	7-14	Present	45-56
*H. diadematus*	1.1-1.8	2	3	47-52	3-4	20	Anterior	Unknown	One anterior & one posterior	9-13	Present	48-53
*H. dimorphicus*	1.1-1.6	0	6	34-36	2-3	18-21	Anterior	Absent	One anterior & one posterior	6-10	Present	36-41
*H. dubius*	1.1-1.3	1	6	31-42	3	14	Anterior	Absent	One anterior & one posterior	10-15	Present	37-44
*H. galeatus*	1.2-1.9	4	3	43-52	5	32-36	Posterior	Present	One anterior & one posterior	10-16	Present	40-52
*H. igualaensis*	1.1-1.5	4	3	40-50	5-6	Unknown	Posterior	Unknown	Both posterior	10-15	Present	36-43
*H. imphalensis*	1.0-1.4	1	3	34-37	3-4	29-30	Anterior	Present	One anterior & one posterior	12-14	Present	37-45
*H. indicus*	1.0-1.6	1	6	33-47	3-4	6-20	Anterior	Present	One anterior & one posterior	8-22	Present	34-42
*H. intermedius*	1.0-1.3	Breaks	3	37-40	4-5	6	Anterior	Unknown	One anterior & one posterior	-	Present	43-47
*H. jalalabadiensis*	1.2-1.7	Breaks	6	37-44	3-4	24-25	Anterior	Present	One anterior & one posterior	16-22	Unknown	-
*H. johani*	1.0-1.4	Not visible	3-6	21-30	3-5	Unknown	Anterior	Unknown	Unknown	14	Present	28-43
*H. maggentii*	1.0-1.4	Absent	3-5	43-48	3-5	Unknown	Anterior	Unknown	Unknown	8-17	Unknown	-
*H. magnistylus*	1.4-2.0	4	3	52-61	4-6	22-34	Posterior	Absent	One anterior & one posterior	12-17	Present	52-58
*H. pararobustus*	**1.2-1.6**	**Breaks**	**3**	**41-44**	**4-5**	-	**Anterior**	**Present**	**One anterior & one posterior**	**9-15**	**Present**	**40-44**
*H. pararobustus*	1.0-1.6	2-3	3	38-49	4-5	18-25	Anterior	Present	One anterior & one posterior	7-15	Present	40-57
*H. puertoricensis*	1.3-1.7	0	5	41-45	3	6-9	Anterior	Absent	Both anterior	19	Unknown	-
*H. puriensis*	1.1-1.3	4	4	33-37	4-5	Unknown	Anterior	Absent	One anterior & one posterior	12-13	Present	31
*H. smokyensis*	0.9-1.8	4	3	41-51	5-6	24	Posterior	-	One anterior & one posterior	-	Present	37-47
*H. sacchari*	1.1-1.2	4	6	34-35	3	8	Anterior	Present	One anterior & one posterior	9-10	Present	39-40
*H. seinhorsti*	**1.3-1.7**	**1**	**5**	**38-44**	**4-5**	**Unknown**	**Anterior**	**Absent**	**One anterior & one posterior**	**13-16**	**Unknown**	-
*H. seinhorsti*	1.1-1.6	1	6	40-49	4	8-12	Anterior	Absent	One anterior & one posterior	10-15	Unknown	-
*H. seshadrii*	1.5-1.8	0	6	42-43	3	20-22	Anterior	Present	One anterior & one posterior	14-18	Unknown	-
*H. singhi*	1.4-2.1	0	3	43-56	4	Unknown	Anterior	Absent	One anterior & one posterior	7	Present	52
*H. stephanus*	1.0-1.5	4	3	43-50	4	24-28	Posterior	Present	One anterior & one posterior	12	Present	30-38
*H. tabacum*	1.3-1.4	1	6	43-45	3-4	Unknown	Anterior	Present	Both posterior	12-15	Unknown	-
*H. tylenchiformis*	0.9-1.4	4	3	42-51	3-4	20-24	Posterior	Present	One anterior & one posterior	8-14	Present	31-38

### Systematics *Hoplolaimus seinhorsti* Luc, 1958

**Figure 1:**
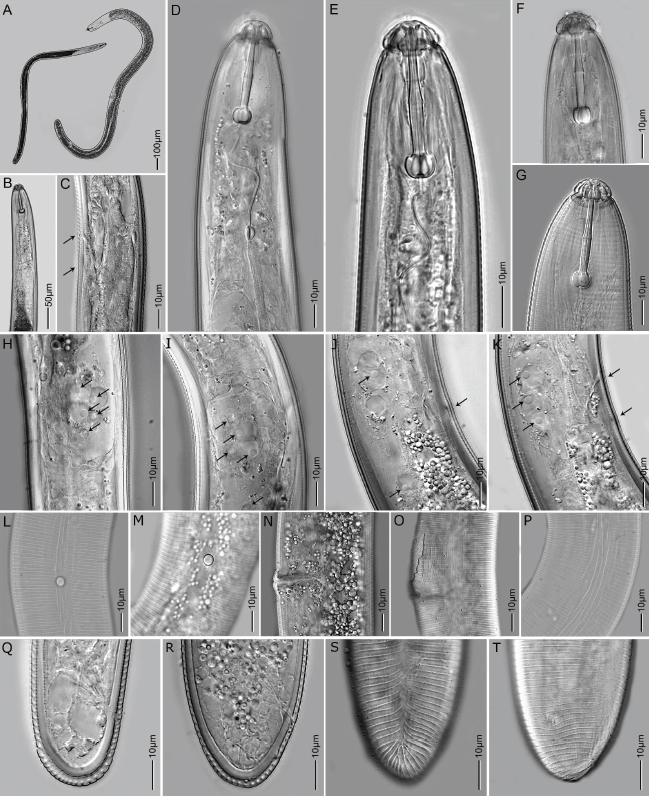
Light microscopy images of females of *H. seinhorsti* (A) whole bodies; (B-G) anterior body in lateral view showing SE pore opening and hemizonid (indicated by arrows), stylet and stylet knobs. DGO and median bulb; (H-K) esophageal region showing five gland nuclei (pointed by arrows); (L,M) scutella, lateral view; (N,O) vulva region in lateral view; (P) lateral incisures around mid-body; (Q-T) tail region showing anal opening, tail annuli number and lateral incisure.

**Figure 2:**
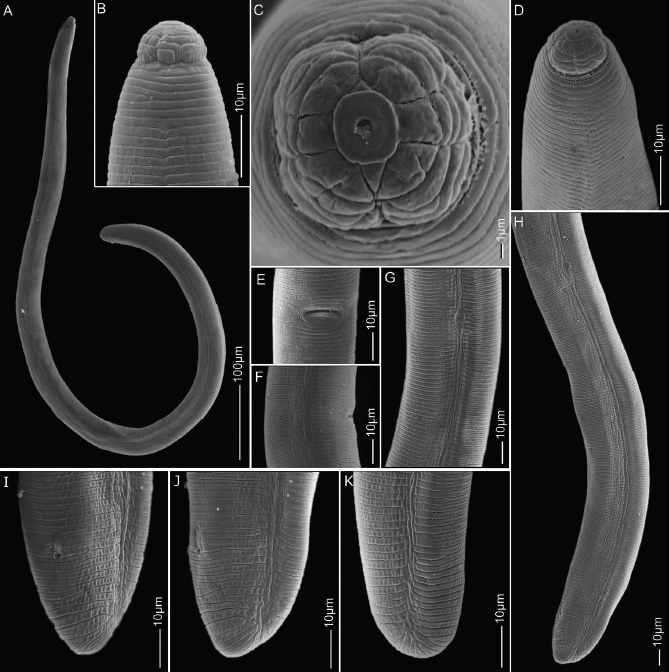
Scanning electron microscopy images of females of *H. seinhorsti* (A) whole body; (B-D) head region; (E,F) vulva region in ventral and lateral views; (G) scutellum anterior to vulva in lateral view; (H) scutellum posterior to vulva along with lateral incisures around the posterior region; (I-K) tails in lateral view showing anal openings.

Figures 1–2, Table 1.

**Table 1.** Morphometric data of *Hoplolaimus seinhorsti* and *Hoplolaimus pararobustus*. All measurements are in μm (except for ratio) and in the form: mean ± sd (range). Species included in this study are presented in the first two columns of the table.

Character	*Hoplolaimus seinhorsti* (Indonesian specimen)	*Hoplolaimus pararobustus* (Nigerian specimens)		*Hoplolaimus pararobustus* (Namibian specimens according to Marais et al., 2020)		*Hoplolaimus pararobustus* (Syntypes; according to Sher, 1963)	
	15♀♀	16♀♀	10♂♂	21♀♀	21♂♂	3♀♀	5♂♂
L	1521±143 (1280-1700)	1322±107 (1161-1552)	1233±50.7 (1171-1325)	1100±76.1 (957-1245)	925±65 (818-1018)	1420-1520	1090-1250
a	26.2±1.3 (22.7-28.3)	29.8±2.9 (24.7-36)	33.8±1.6 (31.1-36.4)	29±3.5 (23.7-36)	29.2±4.2 (22-38.3)	23-25	21-26
b	10.4±0.8 (7.0-11.4)	9.5±0.9 (8-11.4)	10.1±0.5 (9.2-10.7)	7.4±0.7 (6.2-8.6)	6.7±0.4 (6-7.5)	8.8	7-7.4
c	53.7±5.7 (38.1-62.3)	62.2±11.3 (42.6-88.2)	39±1.6 (37-41.6)	59.7±10.5 (46.9-80.6)	34.7±4.3 (25-41)	42-47	28-42
c’	0.8±0.7 (0.7-0.9)	0.7±0.1 (0.5-0.9)	1.49±0.04 (1.4-1.5)	0.8±0.1 (0.6-1.1)	1.5±0.2 (1.3-2)	-	-
DGO	5.3±0.5 (4.6-6.4)	6.6±2.1 (3.7-9.6)	6.2±1.2 (4.5-8.1)	5±0.7 (3-6)	4±0.9 (3-6)	-	-
V	55.2±2.6 (50.2-59.3)	59.4±3.5 (54-70.1)	-	55±2.5 (49-67)	-	57-62	-
Stylet length	42.1±1.7 (38.3-44.1)	42.5±0.8 (41.3-43.8)	42.4±1.1 (41.2-44.3)	36±1.6 (34-40)	34±1.3 (32-38)	46-49	44-46
Stylet cone length	20.8±1.2 (18.2-23.2)	21.7±1.1 (20.2-23.7)	21.3±1.6 (19-23.3)	-	-	-	-
Stylet knob length	7.7±0.4 (6.0-7.9)	7.8±1.1 (5.7-9.1)	5.3±0.7 (4.5-6.3)	6±0.5 (5-7)	5±0.6 (4-6)	-	-
Excretory pore from anterior	151 ±7.91 (140-167)	110±11.8 (85.4-128)	89.2±7.1 (78.3-98.8)	108±11.9 (82-123)	93±13.6 (74-121)	-	-
Nerve ring from anterior	118±4.9 (111-126)	81.1±7.9 (70.8-94.8)	79.8±7.1 (71.6-94.8)	-	-	-	-
Pharynx	-	140±10.7 (114-156)	I23±3.3 (120-129)	-	-	-	-
Pharyngeal gland end from anterior	200±15.7 (162-216)	170±16.3 (143-201)	139±8.1 (131-158)	-	-	-	-
Anterior phasmid of body length (%)	36.1±2.2 (33-38.9)	-	-	-	-	27-30	25-35
Posterior phasmid of body length (%)	81.1±0.9 (80-82)	-	-	-	-	74-79	77-84
Vulva from anterior end	816±72.4 (715-912)	784±61.3 (664-908)	-	-	-	-	-
Diameter at mid-body	58.3±6.1 (44.3-66.6)	44.5±2.6 (39.3-49.9)	36.5±0.8 (35.3-37.6)	39±4 (32-46)	32±2.9 (27-38)	-	-
Anal body diameter	33.8±2.9 (30.1-38.5)	320±2.1 (28.2-37.1)	21.3±0.7 (20.2-22.4)	25±2.2 (21-29)	17±1.3 (15-20)	-	-
Lip region height	9.4±0.7 (8.1-10.4)	7.8±1.2 (6.0-9.7)	8.2±0.5 (7.5-9)	7±0.5 (6-8)	6±0.6 (5-7)	-	-
Lip region diameter	I6.3±0.9 (15.0-18.0)	15.4±1.1 (13.4-17.6)	11.9±1.0 (10-12.9)	13±1 (12-15)	12±0.9 (11-13)	-	-
Lip annulus	-	4.2±0.4 (4-5)	4.1±0.3 (4-5)	-	-	-	-
Tail annuli	13-16	-	-	-	-	-	-
Tail length	28.4±3.1 (23.7-34.9)	21.9±4 (15.7-30.4)	31.6±1.0 (30-32.7)	19±3.1 (15-24)	27±3.5 (22-35)	-	-
Spicule length	-	-	41.4±1.3 (44.7-48.5)	-	38±2.3 (35-42)	-	52-57
Gubernaculum	-	-	21.8±0.7 (19.2-21.8)	-	17±2.3 (11-20)	-	22-26

## Description

*Females:* Vermiform cylindrical body slightly tapering at both the ends. Body slightly curve to open C-shape after fixation. Head with prominent cephalic framework, hemispherical, four labial annuli and distinctly set-off from the body by a deep constriction. In SEM, head region divided into six equal sectors. Irregular longitudinal indentations or striae can be seen on the basal lip annule. Slightly raised ovoid oral disc with a central oral opening. Lateral sectors smaller than the sub-ventral and sub-dorsal sectors and visible amphidial apertures. Lateral field, around the mid-body, four to eight not well-delineated irregular incisures with breaks, and towards the anterior and the posterior regions reduced to one incisures. Stylet strong and large with prominent tulip-shaped knobs. Metacorpus rounded with sclerotized valve. Esophageal glands overlapping the intestine dorsally with five to six gland nuclei. SE-pore at isthmus level anterior to hemizonid and hemizonid about three cuticular annuli long. Two scutella, one anterior to the vulva (about 520 μm from the anterior end) and the other posterior to the vulva (about 1170 μm from the anterior end). Oval vulval opening around mid-body surrounded by unsculptured lips and vulva sometimes appears swollen in live specimens. Posterior epiptygma more conspicuous than the anterior epiptygma. Reproductive system didelphic amphidelphic with two equally developed outreached ovaries, spermathecae round to oval. Tail hemispherical to conoid-hemispherical, 13-16 annuli long.

*Male:* Not found

### Hoplolaimus pararobustus (Schuurmans Stekhoven & Teunissen, 1938) Sher, 1963

Figure 3, Table 1

**Figure 3:**
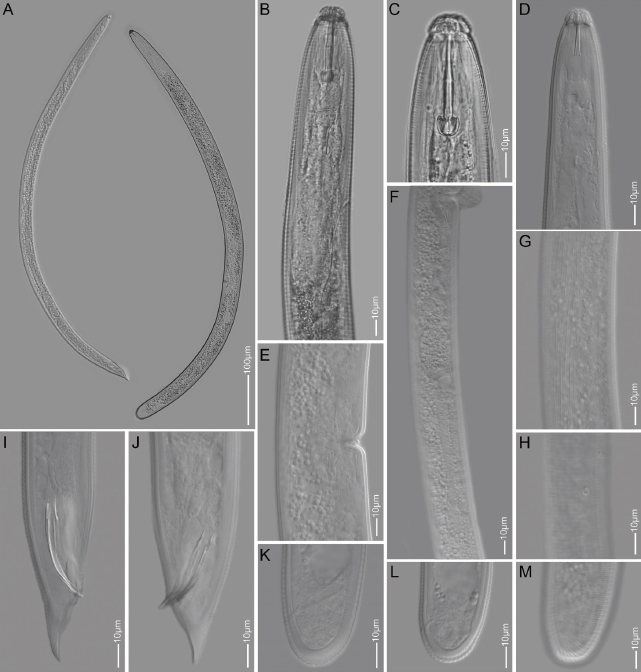
Light microscopy images of females and males of *H. pararobustus* (A) whole body of female and male; (B-D) anterior body in lateral view showing SE-pore opening, stylet and stylet knobs. DGO and median bulb; (E) vulva region with no epiptygma in lateral view; (F) oocytes and ovary with sperm; (G) lateral incisures around mid-body; (H) scutella, lateral view; (I-M) female and male tail region showing anal opening, tail annuli number and lateral incisure.

## Description

*Females:* Vermiform cylindrical body, 1161–1552 μm long, and near C-shape when heat relaxed. Head with prominent cephalic framework, hemispherical, four-five labial annuli and distinctly set-off from the body by a deep constriction. The lateral field is relatively inconspicuous under the light microscope with irregular incisures or broken lines, not well delineated around the mid-body and at the level of vulva and reduced to merely a single incisure towards the posterior part of the body. Stylet strong and large with prominent tulip-shaped knobs. Esophageal glands overlapping intestine dorsally with three gland nuclei. SE-pore above hemizonid and relatively opposite the median bulb. Hemizonid about two to three cuticular annuli long. Two scutella, one anterior to (about 410 μm from the anterior end) and the other posterior to the vulva (about 880 μm from the anterior end). Vulva at 54-70%, reproductive system didelphic amphidelphic with two equally developed outreached ovaries. Spermathecae round to oval with sperm. Tail short (15-30 μm) hemispherical, 13-16 annuli long.

*Male:* Similar to female except for reproductive structures with broad enveloping bursa, and body length generally shorter. Long and prominent spicule and gubernaculum with large and conspicuous bursa extending to the tail tip.

## Molecular characterization

### 28S rDNA

**Figure 4:**
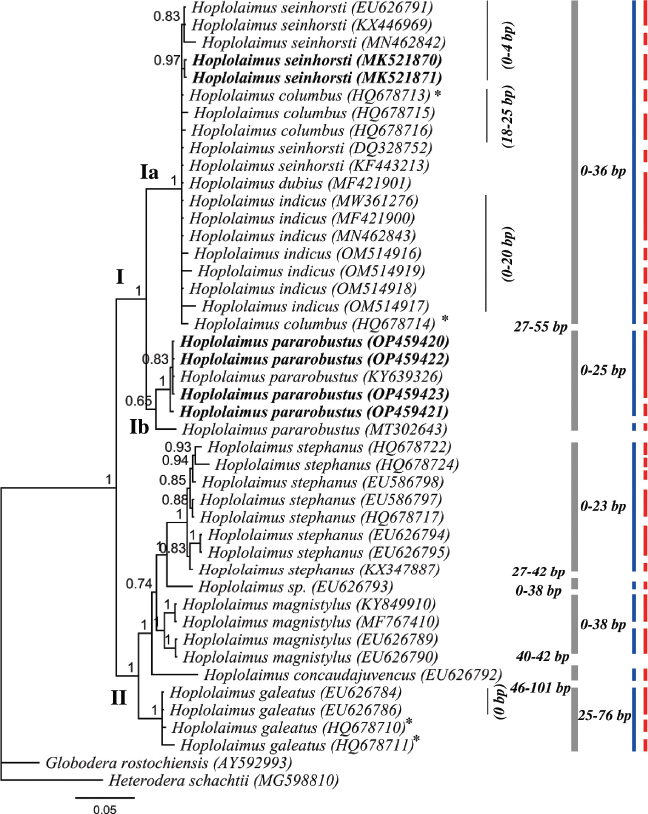
BI phylogenetic tree inferred from analysis of the D2-D3 expansion segment of 28S rDNA sequences from nine known and three unknown *Hoplolaimus* species using the GTR + I + G model. Bayesian posterior probabilities are given next to each node and *H. seinhorsti* and *H. pararobustus* is presented in bold. Intraspecific variation of a clade indicated by a bar is given to the right of the gray bars, nucleotide differences between sister clades is provided left to the gray bars. Red and blue bars represent species boundaries estimated by GMYC and bPTP methods, respectively. A star indicates data with too many non-nucleotide characters in the sequences.

The D2-D3 domains of the 28S rRNA gene alignment (721 bp long) included 43 *Hoplolaimus* sequences and two outgroup species. Two 100% similar sequences of *H*. *seinhorsti* (MK521870, MK521871; 581 & 591 bp long) from Indonesia and four sequences of *H. pararobustus* from Nigeria were generated (OP459420-OP459423; 778 to 779 bp long; 0-3 nucleotides intraspecific variation) (Fig. 4). The *H*. *seinhorsti* sequences in the current study are within a maximally supported clade with *H. indicus* Sher, 1963, *H. dubius* Chaturvedi, Singh and Khera, 1979 and *H. columbus* Sher, 1963; however, without internal resolution. This clade has a sister position to a poorly supported *H. pararobustus* clade. Sequences of *H. seinhorsti* in this study differ by 3-4 nucleotides to the other sequences of *H. seinhorsti* from GenBank (KF443213, DQ328752, MN462842, EU626791, KX446969). Remarkably, our sequences only differ two nucleotides to the sequence of *H. dubius* (MF421901), 2-15 nucleotides to the sequences of *H. indicus* (MW361276, MF421900, MN462843, OM514916, OM514919, OM514918, OM514917) and 5-68 nucleotides different to *H. columbus* sequences (HQ678713-HQ678716). In line with this, the molecular species delimitation results (generalized mixed-yule coalescent - GMYC and Poisson tree process - bPTP) do not provide an unequivocal answer to the species delimitation of the concerning species. The GMYC approach recognized several putative species in clade Ia, although not agreeing with morphologically delimited species; for example our two *H. seinhorsti* sequences are appointed as different from the other *H. seinhorsti* sequences. While, remarkably, the bPTP species delimitation recognized *H. seinhorsti*, *H. dubius*, *H. indicus*, *H. columbus* as one single species.

The *H. pararobustus* sequences generated in this study form a maximally supported clade with *Hoplolaimus* sp. (KY639326) from Kolombia et al. (2017), which is only 0-2 nucleotides different and *Hoplolaimus* sp. (KY639326) is therefore likely also *H. pararobustus*. However, our sequences are 24-25 nucleotides different to the Namibian population of *H. pararobustus* (MT302643) previously characterized by Marais et al. (2020) (Fig. 4). The GMYC approach indicated that the four *H. pararobustus* sequences generated in this study belong to two putative species, despite the fact that all four sequences were from the same population. The bPTP approach indicated the four *H. pararobustus* generated in this study together with *Hoplolaimus* sp. (KY639326) as one species, and the Namibian population of *H. pararobustus* (MT302643) as a different species. For clade II, *H. stephanus* (HQ678722, HQ678724, HQ678717, KX347887) forms a maximally supported clade with *Hoplolaimus* sp. (EU586798, EU586797, EU626794, EU626795) and it is also confirmed by bPTP that these sequences belong to a single species, i.e. *H. stephanus. H. galeatus* (KY849910) forms a maximally supported clade with *H. magnistylus*, also supported by bPTP as being two putative species, and thus one of these species is likely to be mislabeled (Table 4). It is clear that both species delimitation results (GMYC and bPTP) provide highly different outcomes with 27 vs 9 putative *Hoplolaimus* species respectively (Table 3).

**Table 3.** Number of species according to the two species-delimitation methods (GMYC vs bPTP).

Gene region	Number of species				
	Morphospecies (unidentified species included)	GMYC	bPTP
D2-D3 of 28S rRNA	10	27	9
*COI* mtDNA	8	13	13
ITS rRNA	7	16	7
18S rRNA	4	6	5

**Table 4.** List of unlabeled, mislabeled *Hoplolaimus* sequences on GenBank reassigned to corrected species in this study.

			Accession number				
No	Species name on GenBank	28S	*COI*	ITS	18S	Remarks	Reassigned Species name/ Decision
1	*Hoplolaimus* sp.	KY639326	KY639374			Kolombia et al. (2017) provided these sequences as an outgroup, and thus lacking morphological data. The sequence form a maximally supported clade with *H. pararobustus* based on 28S D2-D3 rRNA, the bPTP output also confirmed that this sequence belongs to *H. pararobustus*.	*H. pararobustus*
2	*Hoplolaimus* sp.	EU586798 EU586797 EU626794 EU626795				Bae et al. (2008) provided these sequences. These sequences forms a maximally supported clade with *H. stephanus*, the bPTP output also confirms that these sequences belong to a single species.	*H. stephanus*
3	*Hoplolaimus* sp.				OM218727 OM218726 OM218725	Phylogenetic results and both species delimitation results (bPTP and GMYC) indicate that these sequences and *H. columbus* (KJ934150, KJ934149) sequences belong to a single species.	*H. columbus*
4	*Hoplolaimus* sp.		KP230658 KP230659	KP303683 KP303684		The sequences were originally provided by Holguin et al. (2015), and subsequently associated to the original description of *H. smokyensis* by Ma et al. (2019).	*H. smokyensis*
5	*Hoplolaimus galeatus*	KY849910				This sequence is available on GenBank without morphological data. It forms a maximally supported clade with *H. magnistylus* (MF767410, Donald et al., 2013; EU626789, EU626790, Bae et al., 2008). Assuming that the sequences of *H. galeatus* EU626784, EU626786, HQ678710, HQ678711 are genuine. *H. galeatus* (KY849910) must be incorrectly assigned.	*H. magnistylus*
6	*Hoplolaimus concaudajuvencus*			KP303685 KP303686		Holguin et al. (2015) provided these two sequences as unknown *Hoplolaimus* species in their paper although they were deposited on GenBank as *H. concaudajuvencus*. However, The sequences form a maximally supported clade with *H. magnistylus* (KP303623, KP303634, KP303681, KP303682, EU515325, EU515326, Bae et al., 2008; Holguin et al., 2015), also confirmed by bPTP and GMYC species delimitation.	*H. magnistylus*
7	*Hoplolaimus stephanus*			KP303639		Holguin et al. (2015) provided this sequence as *H. columbus* in their paper, but deposited the sequence on GenBank as *H. stephanus*. This sequence forms a maximally supported clade with other *H. columbus* sequences.	*H. columbus*

### COI mtDNA

**Figure 5:**
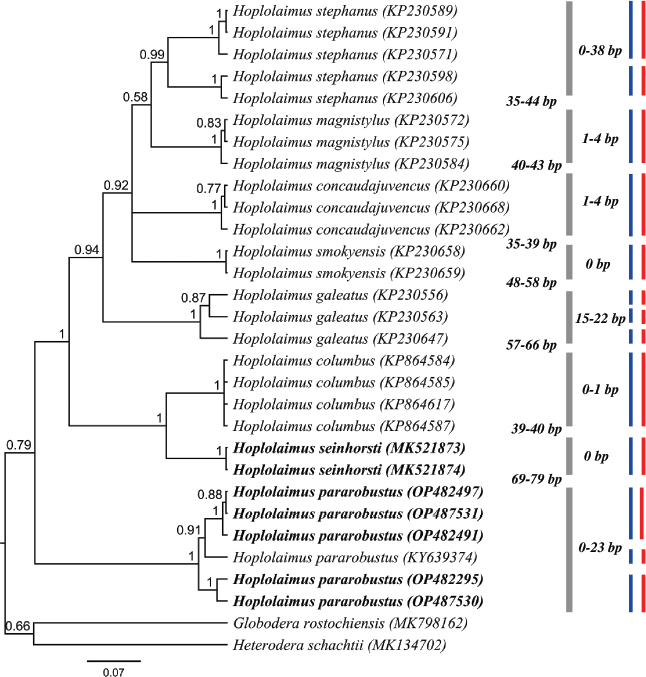
BI phylogenetic tree inferred from analysis of *COI* mtDNA sequences from seven known and one unknown *Hoplolaimus* species using GTR + I + G model. Bayesian posterior probabilities are given next to each node, *H. seinhorsti* and *H. pararobustus* are given in bold. Intraspecific variation of a clade indicated by a bar is given to the right of the gray bars, nucleotide differences between sister clades is provided left to the gray bars. Red and blue bars represent species boundaries estimated by GMYC and bPTP methods, respectively.

The *COI* gene of mtDNA gene alignment (357 bp long) included 26 *Hoplolaimus* sequences and two outgroup species. Two 100% similar sequences of *H*. *seinhorsti* (MK521873, MK521874; 324 & 327 bp long) from Indonesia and five sequences of *H. pararobustus* (OP487531, OP482497, OP482491, OP487530, OP482295; 309 to 353 bp long) from Nigeria were generated. For *H. pararobustus*, intraspecific variation was detected, consisting of 0-19 nucleotides (Fig. 5). The phylogenetic tree inferred revealed a maximally supported sister relationship of *H. seinhorsti* with *H. columbus*, and our *H. seinhorsti* sequences differ by 39-40 nucleotides to *H. columbus* sequences (KP864617, KP864585, KP864584, KP864587). Thus, in contrast to the D2-D3 analyses, *COI* sequences are able to differentiate *H. seinhorsti* and *H. columbus*. However, the results of both molecular species delimitation revealed 13 putative species, which did not correspond to 8 species demarcation based on morphology; only the species delimitation of *H. seinhorsti*, *H. magnistylus*, *H. concaudajuvenis* and *H. colombus* agrees for all approaches.

The *H. pararobustus* sequences generated in this study form a maximally supported clade with *Hoplolaimus* sp. (KY639326) from Kolombia *et al*. (2017), which is 17-23 nucleotides different from the five *H. pararobustus* sequences obtained (Fig. 5). Remarkably, both speciesdelimitation results indicated the five *COI* sequences of *H. pararobustus* of Nigeria and *Hoplolaimus* sp. (KY639374) as three separated species.

### ITS rDNA

**Figure 6:**
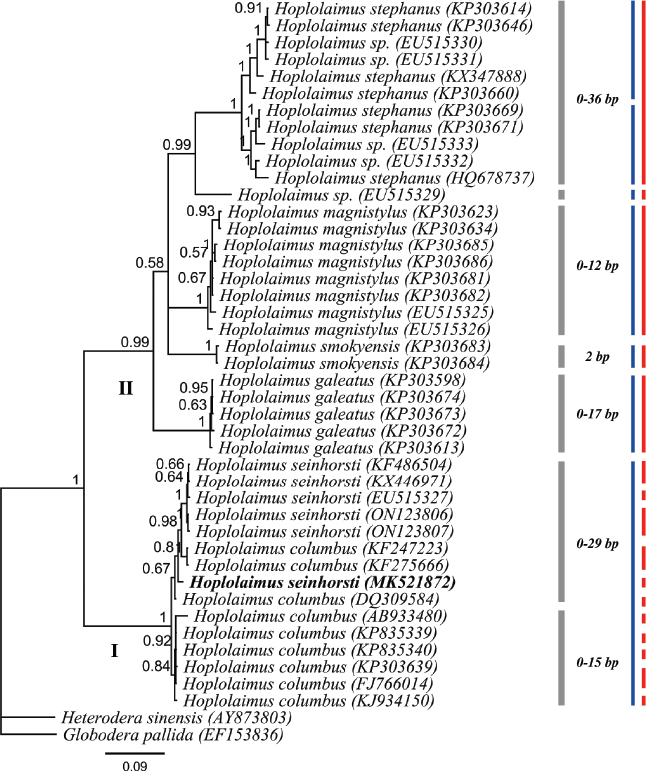
BI phylogenetic tree inferred from analysis of ITS rDNA sequences from seven known and four unknown *Hoplolaimus* species using the GTR + I + G model. Bayesian posterior probabilities are given next to each node and *H. seinhorsti* is provided in bold. Intraspecific variation of a clade indicated by a bar is given to the right of the gray bars, nucleotide differences between sister clades is provided left to the gray bars. Red and blue bars represent species boundaries estimated by GMYC and bPTP methods, respectively.

The ITS rRNA gene alignment (1094 long) included 42 *Hoplolaimus* sequences and two outgroup species. One ITS rDNA sequence was obtained for *H. seinhorsti* from Indonesia (MK521872; 1017 bp long) while ITS sequences for *H. pararobustus* from Nigeria were not obtained. The phylogenetic tree resolved two major clades, and the *H*. *seinhorsti* (MK521872) sequence of the current study is within a maximally supported clade with *H. columbus* and other *H. seinhorsti* sequences from GenBank without internal resolution (Fig. 6). Our *H. seinhorsti* sequence differ by 17-26 nucleotides to the other *H. seinhorsti* (KF486504, KX446971, EU515327, ON123806, ON123807) and 15-25 nucleotides to the sequences of *H. columbus* (KF247223, KF275666, DQ309584, AB933480, KP835339, KP835340, KP303639, FJ766014, KJ934150). The results of both molecular species delimitation approaches showed a high discrepancy, i.e. 16 putative *Hoplolaimus* species based on GMYC vs 7 species based on bPTP (Table 3). For clade II, *H. concaudajuvencus* (KP303685, KP303686) is in a maximally supported clade with *H. magnistylus* (KP303623, KP303634, KP303681, KP303682, EU515325, EU515326) and it is confirmed by bPTP and GMYC that these sequences belong to a single species (i.e. *H. magnistylus*), *H. concaudajuvencus* is therefore mislabeled in GenBank (see Table 4, including further argumentation). Similarly, *H. stephanus* (KP303639) forms a maximally supported clade with *H. columbus* (DQ309584, AB933480, KP835339, KP835340, FJ766014, KJ934150) and this clade is further supported by bPTP as being one putative species (i.e. *H. columbus*). *H. stephanus* (KP303639) should therefore be *H. columbus* (see Table 4, including argumentation). Most remarkably, *H. seinhorsti* and *H. columbus* were, as was the case for D2-D3, delineated as the same species by bPTP, while GMYC delineated them as 11 separate species.

### 18S rDNA

**Figure 7:**
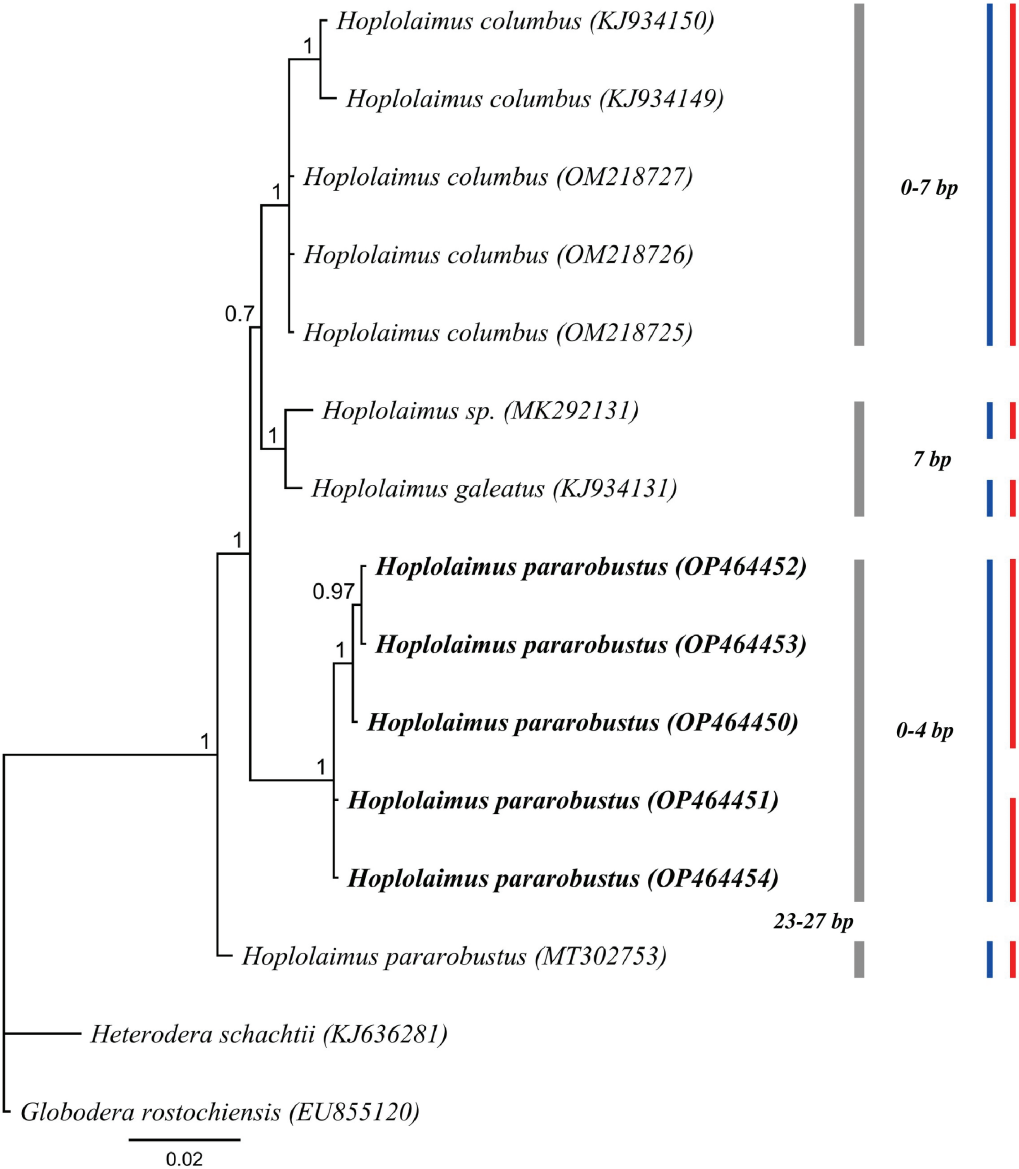
BI phylogenetic tree inferred from analysis of 18S rDNA sequences from three known and two unknown *Hoplolaimus* species using GTR + I + G model. Bayesian posterior probabilities are given next to each node and *H. pararobustus* is given in bold. Intraspecific variation of a clade indicated by a bar is given to the right of the gray bars, nucleotide differences between sister clades is provided left to the gray bars. Red and blue bars represent species boundaries estimated by GMYC and bPTP methods, respectively.

The 18S rRNA gene alignment (923 bp long) included 13 *Hoplolaimus* sequences and two outgroup species. Five new 18S rDNA sequences of *H. pararobustus* were generated from Nigeria (OP464450-OP464454; 879 to 906 bp long) with an intraspecific variation of 0-4 nucleotides (Fig. 7); 18S sequences of *H. seinhorsti* from Indonesia were not obtained. The phylogenetic tree inferred (Fig. 7) revealed a sister relationship of the *H. pararobustus* from Nigeria and all other *Hoplolaimus* species, except *H. pararobustus* from Namibia. Above the different phylogenetic position, the Nigerian *H. pararobustus* sequences were 23 to 27 bp different from that of the Namibian *H. pararobustus* (MT302753). The GMYC and bPTP, species delimitation methods suggested respectively 6 and 5 putative species. Both methods recognized the Namibian population of *H. pararobustus* as a different species, while the bPTP approach recognized all our five *H. pararobustus* sequences as a single species vs two separate species according to GMYC.

## Discussion

*Hoplolaimus seinhorsti* and *H*. *pararobustus* were isolated from the rhizosphere of banana from Indonesia and Nigeria, respectively. The root material of the host plants were not investigated in this study and the damage this nematode species might cause to its host remains to be determined. Nevertheless, both species have already been identified on *Musa* plants; *H. seinhorsti* from banana in India, Martinique and Sri Lanka (Van den Berg 1976; Mukherjee et al., 1983; Larizza et al., 1998; Quénéhervé et al., 2006; Sikora et al., 2018) and *H. pararobustus* from *Musa* plant in several Asian and African countries, including Nigeria (Saeed et al., 1979; Coomans 1983; Fargette and Quénéhervé, 1988; Gowen and Quénéhervé, 1990; Liu and Feng 1995; Vovlas and Lamberti, 1985; Larizza et al., 1998; Speijer et al., 2001; Van den Berg et al., 2003; Loubama et al., 2007; Gaidashova et al., 2009). Other *Hoplolaimus* species have also been found associated with banana (*Musa* sp.), including *H. bachlongviensis* (Nguyen, Bui and Trinh, 2015) from Vietnam (Nguyen et al., 2015), *H. columbus* from Pakistan (Maqbool and Ghazala, 1988; Pathan et al., 2004), *H. indicus* from India and Iran (Maafi and Kheiri 1993; Sundaram 1997; Tilwari et al., 2000; Khan & Hasan, 2010), and other undescribed *Hoplolaimus* species (Khan 1999; Sawadogo et al., 2001; Cannayane et al., 2007; Adriano-Anaya et al., 2008).

It is to be remembered that the results of using morphometrics in species-level identification of nematodes must be examined carefully, as morphometrics of nematodes in general can be influenced by several factors such as environment, host type, geographical origin etc. (Lax et al., 2004; Loubama et al., 2007). In the case of this study, *H. seinhorsti* and *H. pararobustus* could not be unequivocally differentiated by morphometric measurements, but instead with identification being based rather on the number and pattern of lateral incisures, number of labial annuli, number of esophageal gland nuclei, position of SE pore, the absence or presence of an intestinal post-rectal sac and the absence or presence of males. According to Fortuner (1991), the genus *Hoplolaimus* may be divided into two groups based on several phenotypic traits that are either ancestral or derived, including the number of esophageal gland nuclei (3 vs 6), number of lateral incisures (4 vs <4), position of SE pore (below the hemizonid vs above the hemizonid), and the presence of either regular or irregular striae on the basal lip annulus. However, this supposed division between ancestral and derived traits was not reflected in the phylogenetic results obtained in this study.

The D2-D3 of 28S rDNA distinguish between closely-related species in clade Ia (*H. seinhorsti*, *H. columbus*, *H. indicus*, *H. dubius*), a finding that agrees with previous observations (Bae et al., 2008; Ahmadi et al., 2016). For 18S rDNA, the data obtained are too limited to draw clear conclusions. While results based on ITS rDNA agreed fairly well with morphologically-based species delimitation, *H. seinhorsti* and *H. columbus* were not able to be resolved. The current study revealed that only the use of *COI* mtDNA supplied a means of resolving these species, and even then it was only for *COI* that both species-delimitation approaches provided the same output. It is therefore based on this evidence that we propose that the *COI* mtDNA as representing the most suitable barcode region for *Hoplolaimus*, in line with previous observations (Holguin et al., 2015; Ma et al., 2019; Shokoohi et al., 2022).

Remarkably, all phylogenetic and molecular species delineation results indicate that the *H. pararobustus* population from Nigeria and the *H. pararobustus* population from Namibia are not appointed as one single species. However, both populations/species do only differ in morphometrics from each other, the Namibian population has a shorter body length, stylet length and mid-body diameter compared to the Nigerian population. The morphometrics of the Nigerian population are more close to the syntypes according to Sher (1963) and no differences were observed between the *H. pararobustus* population from Nigeria and the type material. This might indicate that the Nigerian population is more likely represents the genuine *H. pararobustus*. However, given the large morphological variation (e.g. the lateral field of the lectotype material ranges from a clear singular line in combination with irregular lines to only unclear irregular lines), it is not obvious to separate the type population and the Nigerian population from the Namibian population on morphological grounds. Therefore, the *H. pararobustus* population from Nigeria and the *H. pararobustus* population from Namibia must at present be considered as cryptic species.

Cryptic species represent a significant component of biodiversity, and are an important factor in quarantine decisions and management strategies (Palomares-Rius et al., 2014). In such cases, if morphological data cannot give a conclusive answer, molecular data of the type specimens are needed, i.e. non-fixed topotype material of *H. pararobustus* (Kanyabayongo, Parc National Albert, Congo; Sher, 1963). This is the only way to conclude the determination of which *Hoplolaimus* population (Namibia vs Nigeria) represents the genuine *H. pararobustus*.

In spite of the increasing use of the coalescence models to study closely-related species that are difficult to differentiate using phenotypic characteristics, these models have only rarely been applied to plant-parasitic nematode investigations (Palomares-Rius et al., 2014; Singh et al., 2021; Nguyen et al., 2022). The present study has investigated putative species boundaries using coalescent-based approaches based on two different models (GMYC and bPTP) and four gene fragments (D2-D3 of 28S, ITS, 18S rRNA and *COI*). Results of these observations show remarkable discrepancies among the genes as well as compared to morphologically-established species (Table 3). Only the *COI*-based results provided identical species delimitation results for both approaches, which is in agreement with the findings of Singh et al. (2021). The GMYC approach revealed many more putative species, while the bPTP is more conservative and agrees better with established species delimitations (Table 3). The GMYC algorithm is based on the time interval to the most recent common ancestor of species and an inherent assumption of monophyly, which is not always the case (Fujisawa et al., 2013), whereas the bPTP algorithm delimits species based on the number of nucleotide substitutions (Prevot et al., 2013). Furthermore, species delimitation methods that are based on single gene trees, for example for the bPTP and GMYC algorithms referred to herein, suffer from serious limitations due to gene tree/species tree incongruence (Zhang et al., 2013). When gene tree topologies are incongruent with one another, it is difficult to determine whether this incongruence is due to incomplete lineage sorting, trans-species polymorphism, hybridisation, or introgression (Leliaert et al., 2014). Therefore, the simultaneous acquisition of several gene sequences will allow for a more precise and substantiated coalescence-based, multilocus species delimitation for plant-parasitic nematodes (Singh et al., 2021). A multilocus approach was not possible in the current study as, at the time of writing, data for only very few multi-loci species are available that have been obtained from the same population, and furthermore, very few such species are associated with both nuclear and mitochondrial sequences.

The findings of this work reinforce the proposals made by Singh et al. (2021) and Nguyen et al. (2022) concerning the need to unambiguously link comprehensive morphological data with both nuclear D2-D3 of 28S rRNA and mitochondrial *COI* gene sequences at the very least. This is clearly particularly necessary for certain species, a case in point being the genus *Hoplolaimus*, an important group of highly damaging plant-parasitic nematodes that were found to display remarkable molecular variations that render their identification especially challenging.
